# Specific Modifications of Histone Tails, but Not DNA Methylation, Mirror the Temporal Variation of Mammalian Recombination Hotspots

**DOI:** 10.1093/gbe/evu230

**Published:** 2014-10-16

**Authors:** Jia Zeng, Soojin V. Yi

**Affiliations:** School of Biology, Georgia Institute of Technology, Atlanta, Georgia Tech

**Keywords:** recombination hotspots, DNA methylation, histone modification, H3K4me3, H3K27me3

## Abstract

Recombination clusters nonuniformly across mammalian genomes at discrete genomic loci referred to as recombination hotspots. Despite their ubiquitous presence, individual hotspots rapidly lose their activities, and the molecular and evolutionary mechanisms underlying such frequent hotspot turnovers (the so-called “recombination hotspot paradox”) remain unresolved. Even though some sequence motifs are significantly associated with hotspots, multiple lines of evidence indicate that factors other than underlying sequences, such as epigenetic modifications, may affect the evolution of recombination hotspots. Thus, identifying epigenetic factors that covary with recombination at fine-scale is a promising step for this important research area. It was previously reported that recombination rates correlate with indirect measures of DNA methylation in the human genome. Here, we analyze experimentally determined DNA methylation and histone modification of human sperms, and show that the correlation between DNA methylation and recombination in long-range windows does not hold with respect to the spatial and temporal variation of recombination at hotspots. On the other hand, two histone modifications (H3K4me3 and H3K27me3) overlap extensively with recombination hotspots. Similar trends were observed in mice. These results indicate that specific histone modifications rather than DNA methylation are associated with the rapid evolution of recombination hotspots. Furthermore, many human recombination hotspots occupy “bivalent” chromatin regions that harbor both active (H3K4me3) and repressive (H3K27me3) marks. This may explain why human recombination hotspots tend to occur in nongenic regions, in contrast to yeast and *Arabidopsis* hotspots that are characterized by generally active chromatins. Our results highlight the dynamic epigenetic underpinnings of recombination hotspot evolution.

## Introduction

One of the most fascinating aspects of mammalian recombination is that recombination events are highly localized in short genomic regions known as “recombination hotspots” (referred to as “hotspots” henceforth) (Steinmetz et al. 1982; Jeffreys et al. 2001, 2004; Arnheim et al. 2003). Moreover, these hotspots change rapidly in evolutionary timescale. Genomic locations of recombination hotspots vary between humans and chimpanzees (Ptak et al. 2005; Winckler et al. 2005), and the signatures of biased gene conversion apparent in human hotspots are not found in homologous regions of closely related primates (Yi and Li, 2005), indicating that hotspots are evolutionarily transient. Moreover, some hotspot locations vary even between individuals (Neumann and Jeffreys 2006; Coop et al. 2008). The observations that hotspots are evolutionarily short-lived, yet remain numerous in mammalian genomes, constitute an intriguing and fundamental question known as the recombination hotspot paradox (Boulton et al. 1997; Pineda-Krch and Redfield 2005; Coop and Myers 2007).

What genomic factors determine the evolution of recombination hotspots? At the level of moderate length genomic sequences (typically hundreds of kilobases), recombination rates correlate with G+C content, gene density, frequencies of simple repeats and transposable elements, and a number of different sequence motifs (e.g., Jensen-Seaman et al. 2004; Meunier and Duret 2004; Groenen et al. 2009). At fine-scale, particular sequence motifs are statistically enriched in recombination hotspots (Bagshaw et al. 2006; Myers et al. 2008). Thus, some sequence characteristics may function as cis-regulators of recombination hotspots. However, DNA sequence itself does not provide a full explanation for variation of hotspots, as numerous nonhotspot genomic regions also harbor similar motifs. The fact that recombination hotspot activities have diverged between humans and chimpanzees, as well as potentially between humans, also indicate a minor role of DNA sequence information in determining the locations of recombination hotspots.

On the other hand, some “epigenetic” factors may regulate recombination hotspots (Paigen and Petkov 2010; Barthès et al. 2011; Smagulova et al. 2011). In particular, patterns of DNA methylation are known to be established at the prophase I in meiosis, which is when recombination occurs (Oakes et al. 2007). Consequently, DNA methylation may affect rates of recombination. Accordingly, Sigurdsson et al. (2009) examined impacts of DNA methylation on variation of human recombination rates. Using methylation-associated single nucleotide polymorphisms (mSNPs) as a surrogate for germline DNA methylation, highly significant positive correlations between mSNP densities and recombination rates at both global- (>500-kb windows) and fine-scale (the proportions of nucleotides belonging to recombination hotspots) were shown (Sigurdsson et al. 2009). However, mSNP densities reflect the frequencies of DNA methylation-caused mutation events, which can be also affected by other factors such as sequence context and repeat occupancy (e.g., Meunier et al. 2005; Elango et al. 2008). Thus it is not clear whether recombination rates covary with the actual DNA methylation levels themselves. Moreover, in light of emerging data on comparative DNA methylation maps of humans and chimpanzees, we can directly test whether the observed rapid divergence of recombination hotspots between humans and chimpanzees is caused by interspecific DNA methylation divergence, as expected if DNA methylation is the underlying driver of recombination hotspot evolution.

In addition, other epigenetic factors, such as modifications of histone tails, may also affect recombination rates. Notably, PRDM9, an important *trans*-acting factor of recombination hotspots in human and mouse genomes (Baudat et al. 2010; Myers et al. 2010; Parvanov et al. 2010) contains a PR/SET domain that is capable of tri-methylation of histone 3 lysine 4 residue. This modification, referred to as H3K4me3, is a major “mark” of active chromatins. The association between recombination hotspots and H3K4me3 is strongly supported by multiple lines of evidence. For example, a study in mouse showed an enrichment of H3K4me3 marks at two specific recombination hotspots, the *Psmb9* and *Hlx1* hotspots (Buard et al. 2009). Changes of PRDM9 sequence caused reconfiguration of recombination hotspots and H3K4me3 modifications (Grey et al. 2011). H3K4me3 is also a potential marker of active recombination sites in yeasts (Borde et al. 2009). Genome-wide mapping of double-strand break (DSB) in mice also reported a significant association between H3K4me3 and recombination hotspots (Smagulova et al. 2011).

It is intriguing that both DNA methylation and H3K4me3 are associated with recombination hotspots, given that H3K4me3, as an active chromatin mark is considered generally antagonistic to DNA methylation (e.g., Mikkelsen et al. 2007). Moreover, recent studies reveal that different types of histone tail modifications could cooccur, and that chromatins often exhibit complex patterns of histone tail modifications that cannot be simply dichotomized as active or recessive states (Ernst and Kellis 2010; Ernst et al. 2011; Comoglio and Paro 2014). Consequently, it is of great interest to investigate how different types of histone modifications vary according to the evolutionary dynamics of recombination hotspots.

Here, we investigate these questions using recently generated DNA methylation data and histone modification data from human, chimpanzee, and mouse genomes. We show that at the global level (at ∼100-kb windows), DNA methylation explains a large amount of variation of recombination rates. However, DNA methylation does not explain the evolutionary variation of recombination at fine-scale or at recombination hotspots. On the other hand, genomic regions enriched in specific histone modification (H3K4me3 and H3K27me3) in human sperms show significant overlaps with recombination hotspots. We also find concordant results in mouse genomes. These novel results indicate that both active and repressive histone modifications may play important roles in shaping the genomic landscape of meiotic recombination hotspots in mammalian genomes.

## Materials and Methods

### Epigenetic Features

Epigenomic data analyzed in this study are summarized in table 1. These include whole-genome methylation maps (“methylomes”) of prefrontal cortex of human and chimpanzee brains (Zeng et al. 2012), and of human and chimpanzee sperms (Molaro et al. 2011) as well as mouse sperms and oocytes (Kobayashi et al. 2012). These methylomes were all generated with next-generation bisulfite sequencing technology and have comparable sequence depths, providing details on DNA methylation at nucleotide-level resolution (table 1). To estimate methylation levels of specific genomic regions, we calculated the mean fractional methylation value for all the mapped cytosines within that region. For each mapped cytosine, the fractional methylation value was calculated as: Total number of “C” reads / (total number of “C” reads + total number of “T” reads), similar to previous studies (Lister et al. 2009; Zemach et al. 2010; Zeng et al. 2012). We also analyzed a genome-wide map of H3K4me3 and H3K27me3 from human sperm (Hammoud et al. 2009) and mouse testis (Cui et al. 2012), as well as H3K4me3 modification map from mouse testis and liver (Smagulova et al. 2011) generated by chromatin-immunoprecipitation sequencing methods. These data provide detailed information on genomic regions enriched in specific histone modifications relative to the genomic background, which were identified using the USeq analysis package (http://useq.sourceforge.net, last accessed October 20, 2014). False-discovery rate correction was performed to provide enrichment significance from a window binomial *P* value.

**Table 1:** Data Sets Used in This Study

Data Type	Reference	Species and Tissue
Genome-wide DNA methylation maps	(Zeng et al. 2012)	Human prefrontal cortex of brain
		Chimpanzee prefrontal cortex of brain
	(Molaro et al. 2011)	Human sperm
		Chimpanzee sperm
	(Kobayashi et al. 2012)	Mouse sperm
		Mouse oocyte
Genome-wide histone modifications maps	(Hammoud et al. 2009)	Human sperm
	(Cui et al. 2012)	Mouse testis
Genetic maps and hotspot locations	(Myers et al. 2005)	Human
	(Auton et al. 2012)	Chimpanzee
	(Brunschwig et al. 2012)	Mouse (SNP)
	(Smagulova et al. 2011)	Mouse (DSB)

### Sequence Features

Custom Perl scripts were used to compute G+C content, normalized CpG (CpG O/E), and CpG dinucleotide count from the human (NCBI 36/hg18) and chimpanzee (CGSC2.1/panTro2) genomes. Proportions of repeats were computed using the *rmsk* table for the location and properties of repeated elements created using RepeatMasker (http://www.repeatmasker.org). Human genetic map and recombination hotspot data were from (Myers et al. 2005). The chimpanzee genetic map and recombination hotspots were based upon the SNP data of ten Western chimpanzees (Auton et al. 2012). Both recombination maps were estimated using the LDhat program. For mouse, two genetic maps were used: One using SNPs from a whole-genome resequencing study of inbred strains (Brunschwig et al. 2012), another from a direct mapping of meiotic DNA DSB that initiate recombination in sperms (Smagulova et al. 2011). To evaluate the overlap between genomic features, we intersected the genomic locations using the liftOver tool from the UCSC Genome Browser. Genomic control regions (for recombination hotspots) are regions randomly sampled (*N* = 10^6^) from recombination hotspot-free whole-genome sequences, while keeping the distribution of genomic length identical to those of actual recombination hotspots.

### Correlation and Linear Regression Analyses

The whole genome was divided into nonoverlapping windows of certain sizes (250 kbs, 500 kbs, and 1,000 kbs). Data were transformed to improve normality using Box–Cox transformation. Linear regression was performed using regional recombination rate as the response variable using a stepwise backward method. Statistical analyses were performed using R package version 2.5.1.

## Results

### DNA Methylation and Recombination Rates Covary at Genome Scale

**Fig. 1.—:**
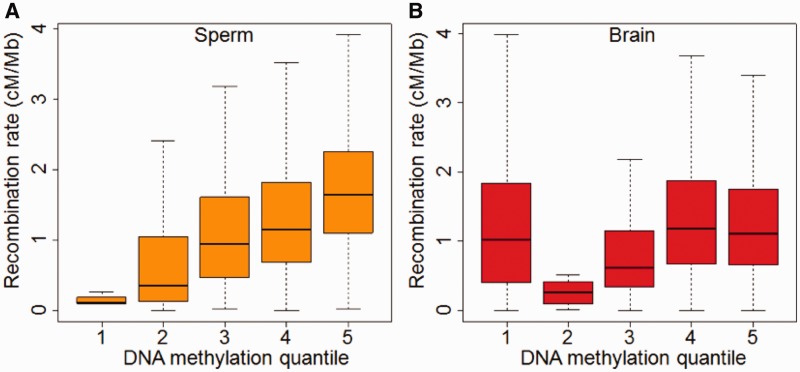
Human recombination rate is positively correlated with DNA methylation in long genomic regions in sperm but not in brain. (*A*) Sperm DNA methylation levels (*x* axis, increases from left to right) correlate with recombination rates (*y* axis) at 500-kb window scale (Pearson’s correlation coefficient *r* = 0.211, *P* < 10^−16^). (*B*) Brain DNA methylation levels and recombination rates at the same scale show at most a weak correlation (Pearson’s correlation coefficient *r* = 0.03, *P* = 0.01).

We first analyzed the relationship between experimentally determined DNA methylation levels and recombination rates in 500-kb nonoverlapping windows in the human genome. Recombination rates increase roughly linearly with sperm DNA methylation levels at 500-kb windows (Pearson’s correlation coefficient = 0.212, *P* < 10^−^^16^; fig. 1*A*). In contrast, recombination rates and DNA methylation levels are at most weakly correlated in brain (Pearson’s correlation coefficient = 0.03, *P* = 0.01). We also performed similar analyses in chimpanzees using the same method. Intriguingly, the correlation in chimpanzee sperms is weak at most, and in the opposite direction to what’s observed in the human sperms (Pearson’s correlation coefficient = −0.04, *P* = 0.002). No correlation was observed in chimpanzee brain (Pearson’s correlation coefficient = −0.002, *P* = 0.84). The significant positive correlations between recombination rates and DNA methylation levels are consistent across different window sizes in human sperm (table 2). On the other hand, these correlations were either not significant or extremely weak in human brains as well as in chimpanzee sperms and brains (table 2).

**Table 2:** Correlation Coefficients between DNA Methylation and Recombination Rate Using Different Window Sizes

Window Size (kb)	Human		Chimpanzee	
	Sperm	Brain	Sperm	Brain
250	0.158***	0.02^NS^	−0.06**	−0.01^NS^
500	0.212***	0.03*	−0.04*	−0.002^NS^
1,000	0.261***	0.04*	−0.01^NS^	−0.007^NS^

Significance: ^NS^*P* > 0.05; *P < 0.05; **P < 10^−6^; ***P < 10^−9^.

The observed correlation between DNA methylation and recombination may be caused by confounding factors that affect both DNA methylation and recombination rates (Meunier and Duret 2004; Elango et al. 2008; Groenen et al. 2009). To examine the effect of DNA methylation on recombination while controlling for other factors, we built a linear model where recombination rates were response variables, and several sequence features (G+C content, number of CpGs, proportion of repeats) and sperm DNA methylation level were predictor variables. The variance inflation factors, which are indicators of multicolinearity among variables, are low (table 3), demonstrating that we could assess individual contributions of each genomic trait without the influence of multicolinearity. We then calculated the standardized coefficients (β), which facilitates an assessment of the strength of association between each predictor variable and the response variable. The linear model could explain over 34% of variability in human recombination rates (table 3). Among the variables included, G+C content and proportion of repeats in the genome window are the strongest and second strongest predictors for the recombination rate (table 3). DNA methylation is also a strong predictor for recombination rates (table 3).

**Table 3:** Multiple Linear Regression Analyses where Recombination Rate Is the Response Variable and Sequence and Epigenetic Features Are Predictors in 500-kb Genomic Windows

	Human		Chimpanzee	
	Standardized β	VIF	Standardized β	VIF
G+C content	0.375***	3.27	0.07*	3.37
Proportion of repeats	−0.237***	1.17	0.006^NS^	1.28
DNA methylation	0.134***	1.42	0.10***	1.47
Number of CpGs	−0.044*	3.59	0.20***	3.92
Adjusted *R*^2^	0.342		0.06	

Note.—VIF, variance inflation factors.

A similar linear model explained much less variation of recombination in the chimpanzee genome (table 3). Notably, the proportion of repeats was not a significant indicator of recombination rates in the chimpanzee genome (table 3). However, given that the significance of repeats on determining recombination rates has been observed in diverse taxa (rodents: [Jensen-Seaman et al. 2004; Shifman et al. 2006], chicken: [Groenen et al. 2009], fly and nematode: [Marais et al. 2001], canine: [Wong et al. 2010], yeast: [Gerton et al. 2000]), this may be due to either incomplete or inaccurate annotations of chimpanzee genomes. Nevertheless, it is notable that DNA methylation is a significant predictor of recombination rates in the chimpanzee genome, when examined in the context of other genomic factors (table 3).

### DNA Methylation Does Not Scale with Variation of Recombination Rates at Fine Scale at Hotspots

**Fig. 2.—:**
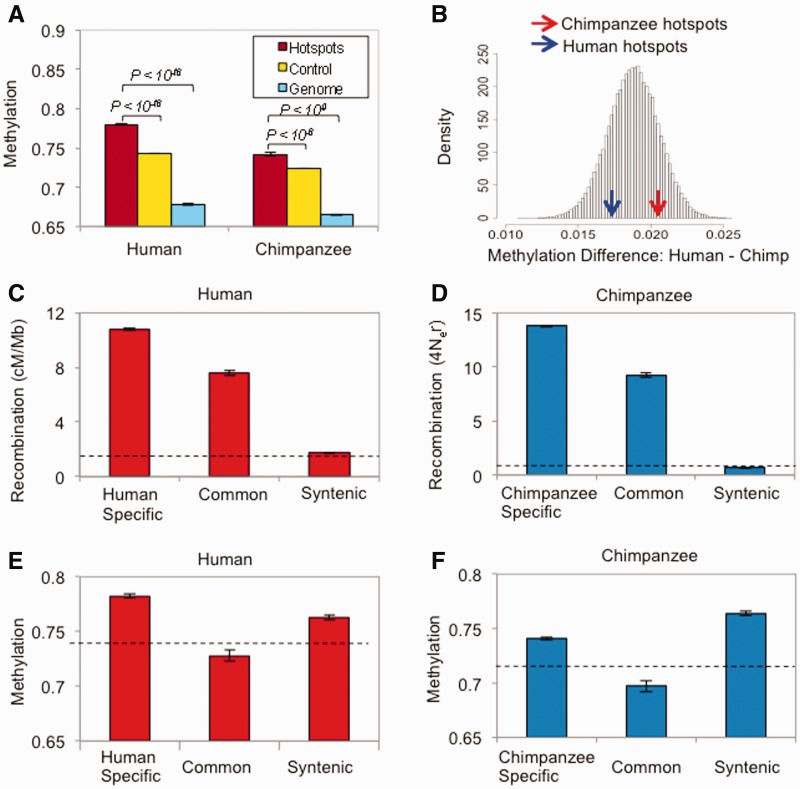
Variation of DNA methylation at human and chimpanzee recombination hotspots. (*A*) Comparison of mean fractional DNA methylation levels between recombination hotspots, genomic control regions (randomly selected regions with the same distribution of lengths and CpG numbers as recombination hotspots, see Materials and Methods), and genome background in human and chimpanzee. (*B*) Distribution of bootstrapped methylation difference (calculated as “Human–Chimp”). The observed interspecies methylation differences at species-specific hotspots are marked (blue arrow for human-specific recombination hotspots, red for chimpanzee-specific recombination hotspots). The observed methylation differences at hotspots do not deviate from the expected methylation differences based upon the bootstrapping. (*C*) Recombination rates at human-specific and common recombination hotspots as well as of the syntenic regions to chimpanzee recombination hotspots. Dotted lines in figure 2*C–F* indicate genomic averages. (*D*) Recombination rates at chimpanzee-specific and common recombination hotspots as well as of the syntenic regions to human recombination hotspots. (*E*) Fractional DNA methylation levels (from sperm) at human-specific and common recombination hotspots as well as of the syntenic regions to chimpanzee recombination hotspots. (*F*) Fractional DNA methylation levels (sperms) at chimpanzee-specific and common recombination hotspots as well as of the syntenic regions to human recombination hotspots.

We then explored whether DNA methylation is associated with fine-scale variation at recombination hotspots. Given the aforementioned positive correlations between DNA methylation and recombination rate in the global scale, DNA methylation level of recombination hotspots should be elevated compared with the genomic background, which is what we observe (fig. 2*A*). The same pattern is observed in the chimpanzee genome, although less pronounced than in the human genome (fig. 2*A*). However, when we examine the relationship between DNA methylation and recombination at recombination hotspots themselves, we find no relationship (supplementary fig. S1, Supplementary Material online). It should be also noted that the observed positive correlations between DNA methylation and recombination at long nucleotides decrease as we examine smaller-scale windows. For example, at 5-kb windows, the correlation coefficient drops to 0.017, although still highly significant due to the large number of windows analyzed (supplementary table S1, Supplementary Material online).

We then examined the temporal and spatial relationship between DNA methylation and recombination hotspots more deeply using data on species-specific and common recombination hotspots. Recombination hotspot locations and usage are highly divergent between humans and chimpanzees (Ptak et al. 2005; Winckler et al. 2005). Accordingly, most of human recombination hotspots in our data set (9,169 of 9,300 hotspots) are specific to the human genome. Likewise, most of chimpanzee recombination hotspots (4,906 of 5,037 hotspots) are chimpanzee-specific. Nevertheless, a total of 131 recombination hotspots are shared between the two genomes, which we will refer to as “common” recombination hotspots. These common hotspots may be those that have existed in the genome of human and chimpanzee common ancestor and have remained as hotspots, or those that have independently arisen in the same positions in the two genomes since their divergence. The latter scenario is highly unlikely, given that hotspots rapidly arise and disappear in the genome, and the target sequence of hotspots are likely to change rapidly due to the fast evolution of trans-regulators such as PRDM9 (Oliver et al. 2009; Thomas et al. 2009; Baudat et al. 2010; Myers et al. 2010; Parvanov et al. 2010). According to the dynamics of hotspot evolution (Pineda-Krch and Redfield 2005; Coop and Myers 2007), strong recombination hotspots are likely to disappear rapidly, whereas weak recombination hotspots may remain longer and shared between these two genomes. Recombination rates of common hotspots are significantly lower than species-specific hotspots in both species (fig. 2*C* and *D*), supporting that the common hotspots are likely to be evolutionarily “older” than species-specific hotspots. Consequently, we can evaluate the evolutionary dynamics of these modifications by comparing epigenetic patterns of species-specific recombination hotspots to those of common hotspots.

We thus compared the variation of recombination and DNA methylation of three types of genomic regions: 1) species-specific recombination hotspots (*n* = 9,169 and 4,906, for humans and chimpanzees, respectively), 2) common recombination hotspots (*n* = 131), and 3) the syntenic genomic regions corresponding to the species-specific hotspots of the other species (e.g., human chromosome regions syntenic to chimpanzee-specific recombination hotspots: *n* = 4,906, chimpanzee genomic regions syntenic to human-specific recombination hotspots: *n* = 9,169).

Although recombination rates follow the expected pattern (species-specific hotspots > common hotspots > syntenic regions, fig. 2*C* and *D*), DNA methylation reveals a very different picture. If recombination rates at hotspots and DNA methylation levels are causatively related, species-specific hotspots should be the most heavily methylated, followed by common hotspots and syntenic regions. However, this is not the case. In the human genome, syntenic regions of chimpanzee recombination hotspots on average exhibit lower levels of DNA methylation than human-specific recombination hotspots, but significantly higher than common recombination hotspots (fig. 2*E*). In the chimpanzee genome, syntenic regions of human recombination hotspots are significantly more methylated than both chimpanzee-specific recombination hotspots and common recombination hotspots (fig. 2*F*). Thus, in both species, DNA methylation levels follow: Human recombination hotspots (or regions syntenic to human recombination hotspots) > chimpanzee recombination hotspots (or regions syntenic to chimpanzee recombination hotspots) > common recombination hotspots.

To examine this observation further, we calculated interspecies methylation differences (as “human DNA methylation level—chimpanzee DNA methylation level” between human-specific recombination hotspots and their syntenic regions in chimpanzee, as well as between chimpanzee-specific recombination hotspots and their syntenic regions in human). We then compared these interspecies methylation differences to those from randomly selected genomic “control” regions (Materials and Methods). Interspecies methylation differences at recombination hotspots do not deviate significantly from the distribution of randomly selected genomic control regions (fig. 2*B*). In other words, divergent recombination hotspots between humans and chimpanzees do not coincide with DNA methylation divergence hotspots between these species. We conclude that the correlation between DNA methylation and recombination rates at a broader genomic scale does not extend to the fine-scale temporal variation of recombination at hotspots.

### Pronounced Histone Modifications at Human Recombination Hotspots

The PRDM9 locus determines a substantial amount of fine-scale recombination rate variation in humans and mice (Baudat et al. 2010; Berg et al. 2010; Myers et al. 2010) and is capable of generating histone modification H3K4me3 (Hayashi et al. 2005). Thus, H3K4me3 profiles in germlines should be correlated with fine-scale recombination rate variation (Smagulova et al. 2011). We examined histone modification profiles of human sperms. Human sperm generally lacks histones, as most histones are replaced with protamines during early germ cell development (Ward and Coffey, 1991; Hammoud et al. 2009). However, a small proportion of genomic regions maintain histone modifications (Hammoud et al. 2009). Specifically, Hammoud et al. (2009) used sequential Micrococcal nuclease digestion and sedimentation to separate chromatin into protamine-bound and histone-bound fractions, and identified genomic regions significantly enriched for histone relative to the input control (total sperm DNA). Histone-enriched regions include several loci implicated in embryonic development.

**Fig. 3.—:**
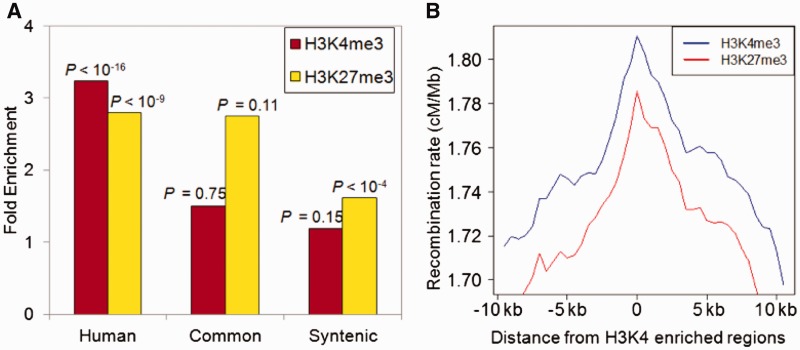
Variation of histone modifications at human recombination hotspots. (*A*) Fold enrichment between observed and expected overlapping of H3K4me3 and H3K27me3 with human-specific recombination hotspots, common recombination hotspots between human and chimpanzee (Common), and regions syntenic to chimpanzee recombination hotspots (Syntenic). (*B*) Mean recombination rate as a function of distance to nearest H3K4me3 and H3K27me3 enriched regions.

We examined whether recombination hotspots show specific enrichments of histone modifications in human sperms. We first examined the H3K4me3 profiles, as this modification has the potential to be directly modified by the PRDM9 protein. We show that, as expected, human-specific recombination hotspots are significantly overrepresented in H3K4me3-enriched regions in sperm: 816 human recombination hotspots overlap with H3K4me3-enriched regions in sperm genomic DNA, whereas the expected number of overlap is 229 (>3-fold enrichment, *P* < 10^−^^16^ by Fisher’s exact test, fig. 3). In contrast, neither common recombination hotspots nor syntenic regions to chimpanzee recombination hotspots exhibit statistically significant enrichment (fig. 3).

We also examined the tri-methylation of H3K27 (H3K27me3), which is another major modification of histone tails, and generally considered as an indicator of “repressive” chromatin. We were motivated by recent analyses of histone modifications demonstrating that different types of histone modifications cooccur in the same genomic regions, and some genomic regions are characterized by both active and repressive marks (e.g., H3K27me3 occurs simultaneously with H3K4me3 at some genomic loci such as some developmental regulators [Mikkelsen et al. 2007; Buard et al. 2009; Hammoud et al. 2009]). We found that the H3K27me3 mark in sperm genomic DNA is also significantly overrepresented in human recombination hotspots (fig. 3*A*). It is also overrepresented (but not significantly so, potentially due to small sample size) in common recombination hotspots, and slightly (1.6-fold) overrepresented at the human syntenic region of chimpanzee recombination hotspots (fig. 3*A*). Consistent with these findings, the average fine-scale recombination rates are elevated around H3K4me3 and H3K27me3 compared with the genomic background (20% and 25% increase at the H3K4me3 and H3H27me3 enriched regions, respectively fig. 3*B*). Moreover, regions harboring both H3K4me3 and H3K27me3 marks in human sperm are also significantly overrepresented in human recombination hotspots (2-fold enrichment, *P* < 10^−^^4^).

### Epigenetic Modifications and Recombination in Mouse Genome

We performed similar analyses in the mouse genome. We first examined the relationship between sperm DNA methylation and recombination rates. Similar to the observation in human genome, recombination rate is significantly and positively correlated with the level of DNA methylation in sperm at the 500-kb genomic windows (Pearson’s correlation coefficient = 0.11, *P* = 1.6 × 10^−^^13^; supplementary fig. S2*A*, Supplementary Material online). Interestingly, DNA methylation and recombination rate is not significantly correlated in mouse oocyte (Pearson’s correlation coefficient = 0.02, *P* = 0.14; supplementary fig. S2*B*, Supplementary Material online), which may be due to the lower resolution of the current mouse oocyte DNA methylome.

We then explored the temporal and spatial variation of fine-scale variation of recombination at hotspots. Unlike human and chimpanzee comparative data, there is no data on epigenetic divergence between closely related mouse species. Nevertheless, two types of mouse recombination hotspot data are available, associated with different evolutionary timescales. One type of hotspot set was identified based upon the genetic map measured by SNPs from inbred mouse strains (SNP hotspots, *N* = 47,068) (Brunschwig et al. 2012). These hotspots are those that have occurred during the genealogy of different mouse strains, thus referred to as “historical” recombination hotspots (Brunschwig et al. 2012). The second data set is hotspots determined from the mapping of meiotic DNA DSB that initiate recombination, which we refer to as DSB hotspots (*N* = 9,874) (Smagulova et al. 2011). This data reflect the “current” recombination hotspots in the specific strain of mouse examined. Thus, comparison of these two data sets may be informative to understanding the temporal and spatial variation of recombination hotspots.

**Fig. 4.—:**
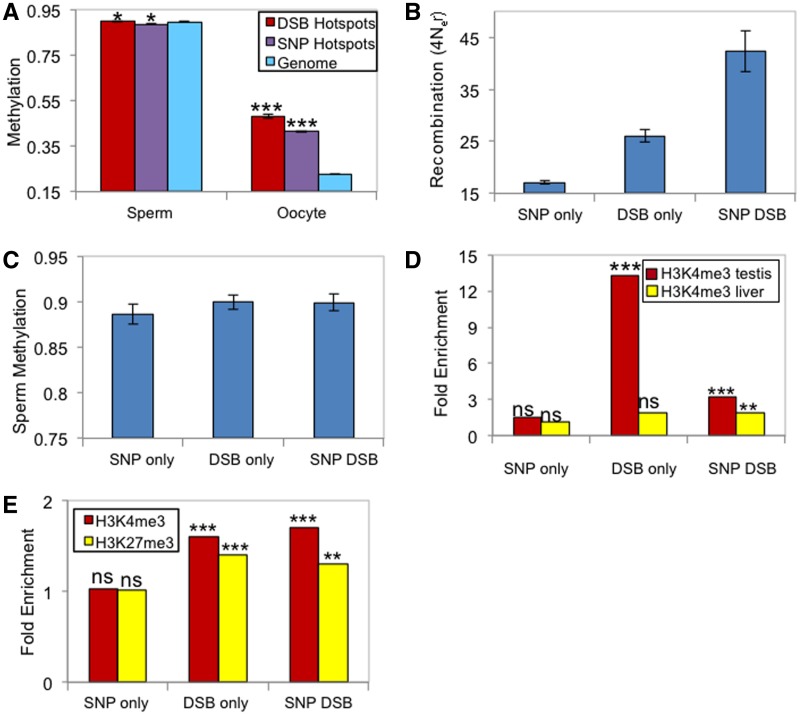
Epigenetic variations at recombination hotspots in mice. (*A*) Comparison of mean fractional DNA methylation levels between DSB-recombination hotspots, SNP recombination hotspots and the genomic background in mouse sperm and oocyte. Significance is assessed by *t* test: *P < 0.05; ***P < 10^−9^. (*B*) Recombination rates at hotspots are highest at hotspots that overlap between DSB- and SNP-study, followed by those identified from the DSB-study only, and the SNP-study only. (*C*) Fractional DNA methylation levels in SNP-only, DSB-only, and SNP- and DSB- study overlap hotspots. (*D*) H3K4me3 fold enrichment at hotspots identified by SNP-only, DSB-only, and by both studies. Significance is assessed by Fisher’s exact test: NS *P* > 0.05; *** *P* < 10^−9^. (*D*) Error bars indicate 95% confidence intervals of the mean.

In mouse sperm, DNA methylation levels at the DSB hotspots are slightly yet significantly higher than the genomic average, while the DNA methylation of the SNP hotspots are slightly lower than the genomic average (fig. 4*A*). It is known that mouse oocytes are significantly hypomethylated compared with the sperms (Kobayashi et al. 2012). Nevertheless, oocyte DNA methylation also exhibits a similar pattern, where DSB hotspots are slightly hypermethylated compared with the genomic background and SNP hotspots (fig. 4*A*).

As expected, SNP hotspots and DSB hotspots show a substantial overlap (2,571 out of 9,874 DSB hotspots are also SNP hotspots, [Brunschwig et al. 2012]). We subsequently classified mouse recombination hotspots into three categories: The SNP only and DSB only recombination hotspots (*N* = 44,498 and 7,303, respectively), which are the hotspots do not have any overlap between the two types; SNP–DSB hotspots (*N* = 2,571), which are the hotspots overlapping between the two data sets. These recombination hotspots should have the following age gradient: DSB only recombination hotspots and SNP–DSB recombination hotspots (younger), and SNP only recombination hotspots (older). According to the observation from the human genome, younger recombination hotspots may exhibit higher recombination rates as well as increased enrichment of H3K4me3 and H3K27me3 modifications than older hotspots.

Consistent with our prediction, we find that recombination rates are highest at SNP–DSB hotspots while SNP only hotspots have the lowest rate (fig. 4*B*). In contrast, DNA methylation levels are statistically not distinguishable among these three types of hotspots (ANOVA, *P* = 0.6, fig. 4*C*), consistent with our finding that DNA methylation variation itself is not a strong indicator of temporal variation of mammalian recombination hotspots.

We then analyzed histone modification data. We first analyzed data on H3K4me3 modification from mouse testis and liver, generated from the Hop2^-/-^ strain (Smagulova et al. 2011). As previously shown (Smagulova et al. 2011), H3K4me3 marks are highly overrepresented in DSB hotspots in testis, but not in liver (fig. 4*D*). H3K4me3 marks in Hop2^-/-^ strain testis are significantly but less strongly enriched in SNP–DSB hotspots, but not in SNP only hotspots (fig. 4*D*). To test whether the observed enrichments hold in a different mouse strain, we analyzed another data sets that are independently generated, namely H3K4me3 and H3K27me3 modification profiles from testes of BALB/c mice (Cui et al. 2012). We found that DSB-only and SNP–DSB hotspots are significantly enriched for both H3K4me3 and H3K27me3 in this data set, whereas SNP only hotspots are not enriched for neither H3K4me3 nor H3K27me3 modifications (fig. 4*E*). We also observe minor but significant enrichment of bivalent chromatin regions in DSB and SNP–DSB recombination hotspots (1.4- and 1.6-fold enrichment, respectively). These observations are largely consistent with the results from the human sperm histone modifications, indicating that specific modifications of histone tails, or combinations of histone modifications, are better indicators of recombination hotspots in human and mouse genomes than DNA methylation.

## Discussion

How do recombination hotspots evolve? One of the most promising answers given so far is that the DNA-binding region of the PRDM9 locus evolves rapidly, thus creating new targets of this trans-factor of recombination hotspots (Coop and Myers, 2007; Oliver et al. 2009; Baudat et al. 2010; Myers et al. 2010). However, sequence motifs may be a necessary condition for some of the hotspots, but not the sufficient answer, as many genomic regions harboring target motifs are not recombination hotspots. In addition, hotspots encoded in identical sequences can exhibit very different recombination activities (Neumann and Jeffreys 2006). Epigenetic factors may be important in the determination of recombination hotspots (e.g., Paigen and Petkov 2010; Barthès et al. 2011). Thus, identifying epigenetic modifications that covary with fine-scale recombination rates will help elucidating the dynamics of recombination hotspot evolution.

Sigurdsson et al. (2009) have reported a genome-wide covariation between DNA methylation and recombination in the human genome. Due to the lack of experimentally determined DNA methylation data at that time, they used mSNPs as a surrogate marker for germline DNA methylation. Our results are qualitatively similar to theirs at the global-(long-range sequence windows) scale. Nevertheless, there are several significant differences between the mSNP study (Sigurdsson et al. 2009) and the current study: 1) mSNPs exhibited generally higher correlation with recombination rates than with the actual methylation data (e.g., correlation coefficient = 0.622 in 500-kb windows from the mSNPs study vs. 0.212 from the actual methylation level in the current study). 2) mSNPs was the strongest predictor of recombination rate in a multiple linear regression model (Sigurdsson et al. 2009), yet DNA methylation level is a weaker predictor than other sequence features (table 3). These discrepancies can be explained by the idea that mSNP density not only reflects DNA methylation levels per se, but also other sequence features that may influence rates of DNA methylation-origin mutations (Meunier et al. 2005; Elango et al. 2008).

Notably, analyses of experimentally determined DNA methylation levels reveal that variation of recombination rates at hotspots may not directly correspond to variation of DNA methylation (fig. 3*E* and *F*), implying that that molecular mechanisms linking recombination and DNA methylation are potentially divergent between fine-scale and broad-scale. To explore this observation further, we utilized the fact that there are common recombination hotspots shared between human and chimpanzee genomes. Given that recombination hotspots evolve rapidly (Myers et al. 2005; Ptak et al. 2005; Yi and Li 2005), these common hotspots are likely to be those that were shared between the two genomes before the evolution of species-specific recombination hotspots. Interestingly, in both species, genomic regions encoding human recombination hotspots exhibit the highest DNA methylation levels, followed by chimpanzee-specific recombination hotspots, and the common recombination hotspots (fig. 2*C*–*F*). These observations suggest that some sequence features can account for the high degree of DNA methylation in both species in spite of highly divergent interspecies recombination rates. Human recombination hotspots, and chimpanzee genomic regions syntenic to human recombination hotspots, may harbor-specific sequence features that are associated with high levels of DNA methylation. Chimpanzee recombination hotspots and human syntenic regions to chimpanzee recombination hotspots also carry some sequence signatures for high levels of DNA methylation. On the other hand common recombination hotspots may not share these sequence features. The nature of such sequence features remains unknown.

The observed correlation between DNA methylation and long-range recombination rate could be due to a third variable, such as histone modification that can interact with both variables and may be more proximal to the cause. The PRDM9 locus encodes a SET-methyltransferase domain in the *Prdm9* gene, which can generate H3K4me3 marks (Hayashi et al. 2005). Studies in specific mouse recombination hotspots (Buard et al. 2009; Grey et al. 2011) and genome-wide patterns in yeast (Borde et al. 2009) strengthen the connection between H3K4me3 modifications and recombination hotspots. We thus investigated the association of the H3K4me3 and recombination hotspots using ChIP-Seq in human sperm (Hammoud et al. 2009) and found that this modification is significantly enriched at human-specific recombination hotspots, but not at common recombination hotspots (fig. 3*A*). Even though the histone modification data are from human sperms rather than testis where meiotic recombination takes place, the consistency between human sperm data and mouse testis data (figs. 3 and 4) indicates that the overrepresentation of H3K4me3 is likely to be a robust trait of recombination hotspots.

Interestingly, we found that H3K27me3 is also enriched in recombination hotspots (fig. 3*A*). In Buard et al. (2009)’ s study of a single recombination hotspot *psmb9* in mouse, H3K27me3 was enriched in “inactive” rather than active recombination hotspots. Our observation is surprisingly at odds with this previous study (Buard et al. 2009). However, these two may not be entirely incompatible, given the vastly different frameworks of these studies (mechanical analyses of a single hotspot versus genome-wide association). H3K27me3 modifications are typically present in polycomb-repressed chromatin (Mikkelsen et al. 2007). Although H3K4me3 and H3K27me3 are generally antagonistic, there are specific genomic regions that harbor both chromatin marks referred to as “bivalent” regions (Bernstein et al. 2006). In the human sperm data, a large number of H3K27me3 marks overlapping with recombination hotspots are found in these bivalent regions. More recently, chromatin state analyses incorporating multiple epigenetic marks have revealed regions that are enriched in both marks in poised promoters or satellite DNA-repressed regions (Ernst and Kellis 2010; Ernst et al. 2011). In addition, H3K4me3 and H3K27me3 are both enriched in mouse recombination hotspots associated with DSBs (fig. 4*E*). These findings indicate that the H3K27me3 mark could also be an important molecular feature at mammalian recombination hotspots and it may affect the recombination pattern simultaneously and interactively with the H3K4me3. Profiling of multiple histone tail modifications in germlines should allow us to investigate the distinctive chromatin states of recombination hotspots more deeply.

Even though germline data from humans are lacking in this respect, there are multiple somatic cells/cell lines where genome-wide distribution of multiple histone modification has been elucidated. We thus examined the B-lymphoblastoid cell chromatin states (GM12878) (Ernst et al. 2011) and the overlaps with recombination hotspots. Among the different chromatin states identified by comprehensive histone modification mapping, state #3 (inactive/poised promoter), states #14/15 (repetitive/CNV) are characterized by the pronounced presence of both H3K4me3 and H3K27me3 marks (Ernst et al. 2011). We found that all these three states exhibit significant overlaps with recombination hotspots (∼2-fold enrichments in all three chromatin states, *P* < 10^−^^16^ by χ^2^ test). In other words, recombination hotspots appear to be also enriched in regions harboring both H3K4me3 and H3K27me3 marks in somatic cells. However, future studies are necessary to examine whether this pattern is inherited through cell divisions from germlines or due to other confounding factors.

The possibility that recombination hotspots are encoded by multiple histone modification signals, and potentially repressive or bivalent markers, could explain several intriguing and currently unresolved observations regarding recombination hotspots in mammalian genomes. First, even though previous studies and our study indicate H3K4me3 overlaps significantly with recombination hotspots, DNA methylation levels of hotspots are also elevated compared with the genomic background (fig. 2). This is at odds with the generally antagonistic associations between H3K4me3 and DNA methylation. However, it may be resolved by recognizing that H3K4me3 at highly recombining regions do not indicate active chromatin state per se, but more ambivalent chromatin states where various histone tail modifications cooccur (Bernstein et al. 2006; Mikkelsen et al. 2007). For example, many genomic regions are found in chromatin states harboring both active and repressive marks (Ernst et al. 2011). The genome-wide correlation between DNA methylation and recombination could indicate the repressive property of highly recombining chromatins. Second, genomic patterns of meiotic recombination vary greatly across taxa (de Massy 2013). In particular, the occurrence of recombination hotspots in mammalian genomes is very different from the patterns in yeast and plants. In yeast, recombination hotspots are enriched in H3K4me3 modification (Borde et al. 2009; Hansen et al. 2011), largely due to the associations between recombination hotspots and promoters (Tischfield and Keeney 2012). Similarly, in *Arabidopsis*, recombination hotspots tend to overlap with active promoters of genes (Choi et al. 2013). Human recombination hotspots, on the other hand, largely avoid genic regions, and are frequently found in subtelomeric regions and positively correlate with repetitive element frequencies (McVean et al. 2004; Myers et al. 2005; McVean 2010). Recombination hotspots in mice also occur in nongenic regions, and the distributions of recombination hotspots can be modulated by manipulating PRDM9 sequences (Brick et al. 2012). The contrasting distributions of yeast and plant recombination hotspots to mammalian recombination hotspots may reflect the differences in chromatin states they represent, the active state represented by the former versus more ambivalent states represented by the latter. This idea could also explain the contrasting relationships between DNA methylation and recombination between mammalian genomes versus fungi and plant genomes. In *Arabidopsis* and fungi, increase of DNA methylation is generally linked to suppression of recombination (Maloisel and Rossignol 1998; Melamed-Bessudo and Levy 2012; Mirouze et al. 2012), whereas hypermethylation is linked to high recombination in mammalian genomes (Sigurdsson et al. 2009 and the current study). These contrasting patterns highlight the dynamic evolutionary interplay between genomic properties and epigenetic properties (Mendizabal et al. 2014). In particular, rapid evolution of epigenetic components and their interaction with meiotic segregation may directly contribute to speciation (Bayes and Malik 2009; Mihola et al. 2009), while shaping the linkage disequilibrium structure at the population level. Elucidating epigenetic mechanisms of recombination hotspot evolution will provide an important model system to investigate how genomic and epigenomic components interact and their evolutionary relevance.

## Supplementary Material

Supplementary tables S1 and S2 and figures S1 and S2 are available at *Genome Biology and Evolution* online (http://www.gbe.oxfordjournals.org/).

Supplementary Data
