# Supplementary material for: Specific Modifications of Histone Tails, but Not DNA Methylation, Mirror the Temporal Variation of Mammalian Recombination Hotspots
Source: Genome Biol Evol. 2014 Oct 16;6(10):2918–29. doi: 10.1093/gbe/evu230 (PMC4224356; doi:10.1093/gbe/evu230)
Supplement: Supplementary Data [file supp_6_10_2918__index.html]

Specific modifications of histone tails, but not DNA methylation, mirror the temporal variation of mammalian recombination hotspots — Specific Modifications of Histone Tails, but Not DNA Methylation, Mirror the Temporal Variation of Mammalian Recombination Hotspots — Supplementary Data 

# Specific Modifications of Histone Tails, but Not DNA Methylation, Mirror the Temporal Variation of Mammalian Recombination Hotspots

## Supplementary Data

files

**Files in this Data Supplement:**

- Supplementary Data - pdf file
- Supplementary Data - docx file
