# Supplementary material for: Specific Modifications of Histone Tails, but Not DNA Methylation, Mirror the Temporal Variation of Mammalian Recombination Hotspots
Source: Genome Biol Evol. 2014 Oct 16;6(10):2918–29. doi: 10.1093/gbe/evu230 (PMC4224356; doi:10.1093/gbe/evu230)
Supplement: Supplementary Data [file supp_evu230_Supplemental_0929.docx]

**Supplemental Information**

**Figure S1: Relationship between recombination rates at recombination hotspots and fractional DNA methylation in human and mouse genomes.**

**
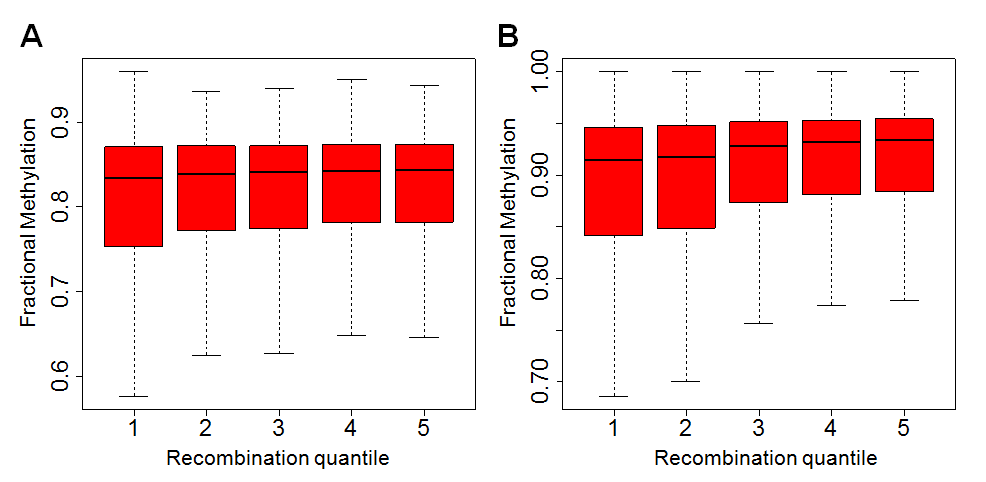
**

(A) Fractional DNA methylation from sperm DNA and recombination rates at recombination hotspots at the human genome. (B) Fractional DNA methylation in sperm DNA and recombination rates at mouse recombination hotspots.

F**igure S2: Genome-wide relationship between recombination rates at recombination hotspots and fractional DNA methylation in mouse.**


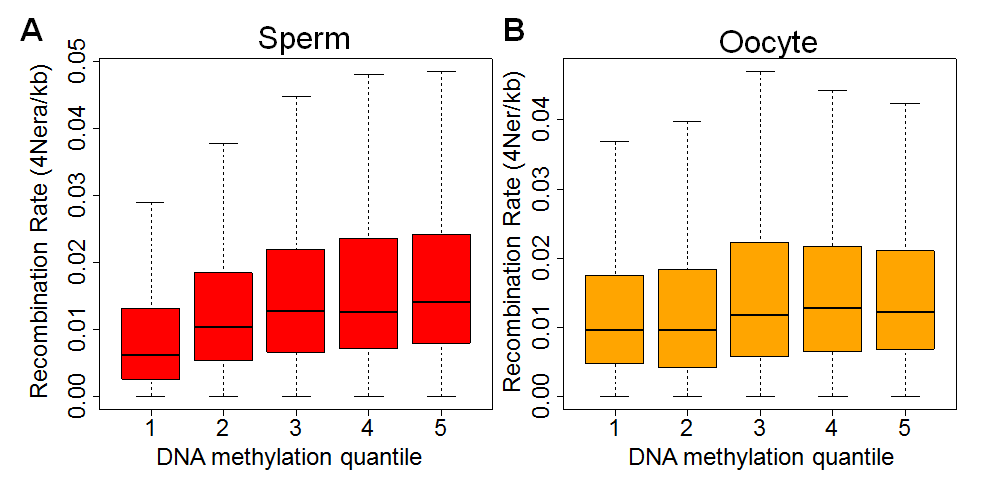


1. Fractional DNA methylation from mouse sperm DNA and recombination rates. (B) Fractional DNA methylation in mouse oocyte DNA and recombination rates.

**Table S1.** Correlation between DNA methylation and recombination in the human genome when assessed using different window sizes.

| Window size (kb) | Correlation | P-value |
| --- | --- | --- |
| 5 | 0.017 | < 2.2e-16 |
| 25 | 0.027 | < 2.2e-16 |
| 50 | 0.059 | < 2.2e-16 |
| 250 | 0.158 | < 2.2 X 10^-16^ |
| 500 | 0.212 | < 2.2 X 10^-16^ |
| 1000 | 0.261 | < 2.2 X 10^-16^ |
