# Supplementary material for: Specific Modifications of Histone Tails, but Not DNA Methylation, Mirror the Temporal Variation of Mammalian Recombination Hotspots
Source: Genome Biol Evol. 2014 Oct 16;6(10):2918–29. doi: 10.1093/gbe/evu230 (PMC4224356; doi:10.1093/gbe/evu230)
Supplement: Supplementary Data [file supp_evu230_Response_to_Reviewers_20140928.pdf]

Response to Reviewers Comments

Reviewer 1's Comments

This paper has a simple, but relevant premise: to identify epigenetic modifications associated with recombination. The authors focus on DNA methylation and two histone modifications (H3K4me3 and H3K27me3), collected from previously generated datasets in human, chimpanzee, and mouse. Of note, they include methylation maps from the brain in addition to reproductive tissues, which is helpful as a control. The role of H3K4me3 in recombination has been studied extensively because of PRDM9, a histone methyltransferase that controls recombination in mice and human, but there is very little data on the role of DNA methylation or H3K27me3 in controlling recombination. Furthermore, with methylation and recombination data available for both human and chimpanzee, the authors are able to look for changes in epigenetic profiles associated with shifts in recombination hotspots, a very interesting idea. They identify broad scale associations between recombination rate and DNA methylation in human, but do not observe these associations at fine scales. This is in contrast to results from other organisms (chiefly Arabidopsis), in which DNA methylation represses recombination. They detect overlap between both H3K4me3 and H3K27me3 and recombination hotspots, surprising since H3K4me3 is an active chromatin mark, while H3K27me3 is a repressive chromatin mark. This point especially calls for further investigation.

I had several questions regarding the datasets used. First, I would like to see the resolution of each dataset used for epigenetics (are they base pair resolution?) included in the Materials & Methods.

Response: DNA methylation maps are generated by whole genome bisulfite sequencing, which provides nucleotide-resolution information. Histone modification data are processed to generate enrichment statistics for specific genomic regions. We provided these details in the 'Epigenetic Features' section in the Materials and Methods.

The authors mention that human sperm generally lack histones, but that a small proportion of genomic regions maintain histone modifications – what regions are these? Does this bias the dataset in some way?

Response: Hammoud et al. (2009) used sequential Micrococcal nuclease (MNase) digestion and sedimentation to separate chromatin into protamine-bound and histone-bound fractions, and identified genomic regions significantly enriched for histone relative to the input control (total sperm DNA). These regions were enriched in genic regions compared to non-genic regions (FDR < 0.0001), especially for loci important for embryonic development. We examined whether the histone-bound

fractions provided in this paper is enriched for recombination hotspots and did not observe any over-representation of recombination hotspots in total MNase fractions. The manuscript is updated with these details.

With mouse, I'm curious to know how much epigenetic variation exists between strains, e.g. can you use methylation from one strain with recombination data of another strain?

Response: Currently the information on strain-specific variation of epigenetic marks is lacking. Nevertheless, there are two mouse testis histone modification data sets, and we tried to assess the impact of specific data set on our analyses as follows. The data we used for mouse histone modifications come from Cui et al. (2012), where both H3K4me3 and H3K27me3 data were generated. The only other paper that has specifically examined potentially germline specific histone modification data come from Smagulova et al. (2011) where only H3K4me3 was analyzed from mouse testis. To test whether strain-specific histone modification patterns may affect our results, we re-analyzed Smagulova et al.'s data. This analysis shows that DSB hotspots are highly enriched in H3K4me3 in testis, but not in liver, a finding consistent with Smagulova et al's original publication. In addition, we observe that H3K4me3 is also enriched in SNP-DSB hotspots but not in SNP only hotspots, which is consistent with the results from Cui et al's data. We have updated the manuscript with the new analysis (p. 14 of the revised manuscript and the updated Figure 4).

I was curious as to how the scales of analysis were chosen. The analysis of a correlation between recombination and DNA methylation jumps from 250kb down to discrete loci (hotspots, typically 1-2 kb). It would be interesting to see at which scales the association persists (10kb, 5kb?).

Response: The observed correlations between DNA methylation and recombination decreases quickly as the window sizes become smaller, as shown in the table below. We have updated the manuscript with this information (p. 9 of the revised manuscript and a new Table S1).

**Window size (kb)**	**Correlation**	**P-value**
5	0.017	< 2.2e-16
25	0.027	< 2.2e-16
50	0.059	< 2.2e-16
250	0.158	$< 2.2 \times 10^{-16}$
500	0.212	$< 2.2 \times 10^{-16}$
1000	0.261	$< 2.2 \times 10^{-16}$

I’d also like to see some discussion as to what causes this broad scale association.

Response: The hypotheses we mention in the manuscript include 1) the possibility of similar sequence determinants, affecting both DA methylation and recombination (such as repeat sequences), and/or 2) the possibility that recombination events are largely associated with ‘ambivalent’ chromatin states, where the ‘repressive’ marks co-occur with DNA methylation. However, needless to say, much more research is necessary to elucidate the exact mechanisms.

Is there an issue of power in detecting features at hotspot/syntenic regions vs. common hotspots?

Response: Even though the number of common hotspots is smaller than the numbers of species-specific hotspots and syntenic regions, we do not feel that this is the reason underlying the observed patterns, because 1) rates of DNA methylation and recombination all show very small confidence intervals (Figure 2), and 2) when we performed random sampling of human and chimpanzee hotspots while controlling for the distribution of CpG dinucleotides, the pattern remained (Figure 2B).

One explanation I could think of for the observed methylation patterns around hotspots involves GC biased gene conversion. This process could increase the amount of cytosines and CpG sites (and thereby sites of possible DNA methylation) as more recombination occurred. Perhaps the common hotspots have experienced the least amount of recombination over time, therefore experiencing less GC biased conversion? This is an off the cuff idea, but perhaps GC biased gene conversion should be discussed in this context.

Response: There are much confounding associations between GC contents, CpG contents, DNA methylation, and recombination (e.g., Elango et al. 2008). It is worth noting that the effect of DNA methylation on recombination is independent of CpG contents and GC contents, at least in the context of our statistical analysis in Table 3. It is also relevant to note that GC contents and DNA methylation generally have negative relationships in the human genome, and the most highly CpG rich regions of the human genome, or CpG islands, are devoid of DNA methylation. These sometimes counteracting associations indicate that the exact relationships between DNA methylation, GC content evolution and recombination are non-linear, and we hope to address these issues more fully in a separate study, as we found it very difficult to incorporate these points in the manuscript without diffusing the main focus.

Throughout the paper I found myself wondering why the mark H3K27me3 was included in the analysis. There was no discussion of its function, and I knew nothing of it except that it generally served a repressive role. There is a nice and interesting explanation in the discussion (p. 16), so I feel

it should be brought up in the Introduction and Results for readability and clarity.

Response: We have updated the results section to discuss the potential significance of H3K27me3 (p. 12).

Along these lines, the finding that both H3K27me3 and H3K4me3 are enriched in hotspots is very interesting. I would like to see more analysis on this subject: How often are they simultaneously enriched at hotspots? Are there hotspots enriched exclusively for one and not the other? Are there any differences in the hotspots enriched in one but not the other (sequence features, proximity to genes, etc.)? Similarly, I think p.16 lines 46-51 should be moved to the results and clarified (as written is a little confusing).

Response: Following the reviewer's suggestion, we have updated the manuscript and put the results on bivalent regions more clearly in the Results section. We are very interested in analyzing different genomic regions according to their chromatin states in germlines. However the data on these aspects are still lacking and we hope to address these questions directly in a near future (i.e. we are preparing a grant submission to examine these specific questions).

In general, references are outdated. There are a lot of papers about DNA methylation and recombination, PRDM9, H3K4me3, etc. in the past couple years that should be read and incorporated and/or cited. In particular, the authors should comment on the pattern of methylation and recombination in other organisms and why they might differ from human. For example:

Brick K., Smagulova F., Khil P., Camerini-Otero R. D.,Petukhova G. V., 2012 Genetic recombination is directed away from functional genomic elements in mice. Nature 485: 642-645.

Grey C., Barthes P., Chauveau-Le Friec G., Langa F., Baudat F., et al., 2011 Mouse PRDM9 DNA-Binding Specificity Determines Sites of Histone H3 Lysine 4 Trimethylation for Initiation of Meiotic Recombination. Plos Biology 9.

Maloisel L.,Rossignol J. L., 1998 Suppression of crossing-over by DNA methylation in Ascobolus. Genes Dev 12: 1381-1389.

Choi K., Zhao X., Kelly K. A., Venn O., Higgins J. D., et al., 2013 Arabidopsis meiotic crossover hot spots overlap with H2A.Z nucleosomes at gene promoters. Nat Genet 45: 1327-1336.

Melamed-Bessudo C.,Levy A. A., 2012 Deficiency in DNA methylation increases meiotic crossover

rates in euchromatic but not in heterochromatic regions in Arabidopsis. Proc Natl Acad Sci U S A 109: E981- 988.

Mirouze M., Lieberman-Lazarovich M., Aversano R., Bucher E., Nicolet J., et al., 2012 Loss of DNA methylation affects the recombination landscape in Arabidopsis. Proc Natl Acad Sci U S A 109: 5880-5885.

Yelina N. E., Choi K., Chelysheva L., Macaulay M., de Snoo B., et al., 2012 Epigenetic remodeling of meiotic crossover frequency in Arabidopsis thaliana DNA methyltransferase mutants. PLoS Genet 8: e1002844.

Response: We have updated the references and added discussion on species differences (although the previous version already included Choi et al. manuscript and some discussion on species differences).

Overall, there are some interesting findings here. I thought the detailed analysis of differences between the Sigurdsson paper and this paper was overkill – perhaps a reduction of this issue in the introduction or discussion and more focus on an interpretation of the authors' own findings would improve this manuscript.

Response: We agree that this section is now reduced.

Minor comments

p. 3 line 57 – p. 4 line 5 Rephrase

Response: now rephrased to improve clarity.

p.8 lines 36-45 I had to read this several times and I still find it confusing. Please clarify.

Response: now rephrased to improve clarity.

p. 17 line 27-31 What does this mean? Please elaborate

Response: now rephrased to improve clarity.

Reviewer 2's Comments

In the manuscript by Yi and Zeng the authors have nicely taken advantage of published methylome and epigenomic maps to address the question of the importance of DNA methylation and histones marks for the location of recombination hotspots in mammals. Conversely to previous studies the authors have focused on testis, sperm and ovocytes epigenomic maps trying to have a more direct evidence of the possible impact of epigenetic marks on the recombination map. Their results show that epigenetic marks have a distinct influence at large scale versus fine scale. Furthermore they show that bivalent marks (H3K4me3 and H3K27me3) are enriched at hotspots.

The discussion is very well written and interesting; it highlights differences on the impact of the epigenome on recombination landscapes between different models. Here some recent literature in Arabidopsis could be cited for readers not familiar with plant models (e.g. Choi et al Nature Genetics 2013, see also Henderson Curr Op Plant Biol 2012 for more details).

This study shows that public epigenomic datasets can be smartly used and combined to answer important biological questions. I enjoyed reading this manuscript and I believe it will be of high interest to GBE readers.

Response: We truly appreciate the positive comments. We have updated the citations to include analyses of plant models.

Reviewer 3's Comments

This is a very thorough and interesting study linking epigenetic variation to recombination activity in the human genome. The use of the human and chimp datasets for DNA methylation (determining species specific, common, and syntenic regions) is a particularly strong part of the study. I have only a few questions regarding this study:

1. What is the significance of recombination hotspots correlating with bivalent chromatin? These regions normally are seen to have a role in imprinting and development. Do human recombination hotspots also correlate with bivalent chromatin in somatic cells? I would assume that these cells would have fewer bivalent regions (since they are differentiated and developed cells) and it would be interesting to see how this would affect the hotspot landscape, regulation, and evolution. If this cannot be addressed with the currently available data, can the authors speculate about this point in the Discussion?

Response: This is an excellent question, however the lack of data prohibits us from performing a comprehensive analysis. For example detailed histone tail modification data from human testis or

ovary are conspicuously lacking, and data from somatic cells/cell lines are also limited (more data are available for cancer cells, but they are not relevant for our goals). Nevertheless we examined published somatic cell data to determine whether the distribution of recombination hotspots show the expected pattern. Specifically, we analyzed B-lymphoblastoid cell chromatin states from Ernst et al. (2011). This analysis provided results that are generally consistent with our manuscript (chromatin states with marked increase of H3K4me3 and H3K27me3 significantly overlap with recombination hotspots). However we refrain from extending this finding given the limitation of data and control. Nevertheless we believe including this analysis has improved the contents of the manuscript (p. 18 of the revised manuscript) and thank the reviewer for the suggestion.

2. Can the authors highlight their results in the Discussion more, especially in terms of species evolution and variation?

Response: We have expanded on the discussion highlighting the differences between species on the relationship between recombination and epigenetic factors.

Small points:

- The suggested links to the datasets did not work.

Response: The only link that didn't work is recombination rate data from Myers et al. 2005. We have previously used the link provided by Myers et al. 2005 paper (in their citation #8) and downloaded the data. In the revision process we checked and found that this link doesn't work anymore. We have contacted the first author but haven't heard back. We hope the authors of the original publication will update their link soon. However given the time constraint we have in our revision, we removed the links from the revised Table 1.

- Figure 2:B: hard to see the arrows in the distribution. I don't know if that is just a resolution issue as this is not the final copy. C-F: what are the dotted lines? Genomic level?

Response: We updated the Figure 2B to more clearly show the arrows. We also made sure the figures have enough resolution. The dotted lines in Figures 2C-F indicate genomic averages. We realized there were some errors in the line in Figure 2C in the previous version and corrected it.

- Figure 4: Would like to see some P-values for A. They say they are significant but they look similar Having P-values would make a stronger case here.

Response: P-values are now indicated and the figure legend is updated.
